# Immunostaining for p53 and p16^CDKN2A^ Protein Is Not Predictive of Prognosis for Dogs with Malignant Mammary Gland Neoplasms

**DOI:** 10.3390/vetsci6010034

**Published:** 2019-03-25

**Authors:** John S Munday, Harsha Ariyarathna, Danielle Aberdein, Neroli A Thomson

**Affiliations:** Pathobiology, School of Veterinary Science, Massey University, Palmerston North 4442, New Zealand; h.ariyarathna@massey.ac.nz (H.A.); d.aberdein@massey.ac.nz (D.A.); n.thomson1@massey.ac.nz (N.A.T.)

**Keywords:** canine mammary gland neoplasia, immunostaining, p16, p53, prognosis

## Abstract

Mammary gland tumors (MGTs) are common in dogs and show a variable clinical behavior that is difficult to predict. Currently, few immunohistochemical markers have been established to predict the prognosis of a canine MGT. However, p53 immunostaining has been variably reported to be prognostic for canine MGTs. Additionally, while p16^CDK2NA^ protein (p16) immunostaining has been found to be prognostic for human breast cancers, this marker has never been evaluated as a prognostic marker for canine neoplasms. In the present study, the prognostic utility of p53 and p16 was evaluated in 35 canine malignant MGTs. It was observed that 19 (54%) dogs died due to their MGTs with an overall mean survival time (MST) of 882 days. Seven MGTs showed p53 immunostaining, but this was not significantly associated with death (4 of 7 vs. 15 of 28; *p* = 0.6) or MST (670 vs. 934 days; *p* = 0.57). Five dogs had MGTs with no p16 immunostaining, 28 MGTs had intermediate p16 immunostaining, and two MGTs had increased p16 immunostaining. Neither death due to MGT (4 of 5, 14 of 28, or 1 of 2; *p* = 0.28) nor MST (683, 927, and 307 days; *p* = 0.31) were significantly associated with p16 immunostaining. Interestingly, p53 immunostaining was significantly associated with an increase or loss of p16 immunostaining. This is the first time that p16 has been evaluated as a prognostic marker for canine neoplasms. While these results suggest that a proportion of canine MGTs develop by cellular mechanisms that alter both p53 and p16 expression, there was no evidence that defects in p53 or p16 alter the behavior of a MGT. Neither p53 nor p16 was found to significantly predict prognosis, although this could reflect the limited number of MGTs included in the study.

## 1. Introduction

Mammary gland tumors (MGTs) are common in intact female dogs, and 40%–50% of these neoplasms are malignant [1]. The biological behavior of malignant MGTs varies from slow invasion of surrounding tissue to rapid metastases. Due to this inconsistent biological behavior, dogs with malignant MGTs have widely variable survival times that range from a few months to many years [2]. Currently, histological subtype is considered the best way to predict the behavior of a canine malignant MGT [2]. However, in both dogs and humans, there has been considerable interest in the use of immunohistochemical markers to more accurately predict neoplasm behavior.

One marker that has previously been evaluated in human and canine MGTs is p53. The p53 protein is considered to be the ‘guardian of the genome’ as it is responsible for detecting DNA damage and triggering apoptosis of damaged cells. Despite the critical role of this protein in cancer biology, studies in humans have typically found only weak associations between prognosis and p53 expression within breast cancers [3]. In dogs, significant differences in p53 immunostaining were reported between benign and malignant canine MGTs [4]. Furthermore, increased p53 immunostaining was significantly associated with a less favorable prognosis in a series of 10 dogs with malignant MGTs [5]. In contrast, p53 immunostaining was not significantly associated with survival in a series of 40 dogs with a mixture of benign and malignant MGTs [6]. A potential disadvantage of p53 immunostaining is the difficulty of interpreting an absence of immunostaining within the cells. While positive immunostaining indicates the presence of missense mutations that have resulted in the accumulation of non-functional p53 proteins, the absence of p53 immunostaining could either indicate normal p53 function or a complete lack of p53 protein due to the presence of nonsense mutations in the p53 gene [7].

The p16^CDKN2A^ protein (p16) is a tumor suppressor protein that prevents cell cycling by regulating the function of the retinoblastoma (pRb) protein [8]. Cells normally contain scant to moderate cytoplasmic immunostaining. However, if a cell cannot produce pRb, this results in a marked increase in p16 within the cell as well as the promotion of uncontrolled cell replication [9]. Mutations within the p16 gene also reduce the ability of the cell to prevent cell cycling but decrease p16 immunostaining. The use of p16 as an immunohistochemical marker to predict cancer prognosis is best illustrated in human oral squamous cell carcinomas (SCCs)with increased p16 immunostaining being the strongest predictor of a favorable prognosis for these cancers [10]. While less research has been done on the prognostic utility of p16 immunostaining in human breast cancers, increased p16 immunostaining has been associated with a more aggressive cancer phenotype and shorter survival times in several studies [11,12]. In dogs, variable p16 immunostaining has been previously reported in MGTs [13], although to the authors’ knowledge, the use of p16 as a prognostic marker for canine MGTs has not been previously investigated.

Due to current uncertainty regarding the prognostic significance of p53 immunostaining for canine MGTs, the ability of p53 immunostaining to predict prognosis was evaluated in a series of canine malignant MGTs. Additionally, the association between p16 immunostaining and prognosis was also investigated in these canine MGTs. This is the first time that p16 immunostaining has been evaluated as a prognostic marker for a canine neoplasm.

## 2. Materials and Methods

The databases of two diagnostic laboratories (New Zealand Veterinary Pathology Ltd. and IDEXX New Zealand Ltd.) were searched for cases of canine malignant MGTs for which a diagnostic sample had been submitted for histology. Cases that had been received during 2012–2015 were selected, and surveys were sent to the submitting veterinarians in January 2018. Information requested on the surveys included the treatments that had been used, whether or not the dog had died due to the MGT, and the survival time of the dog. Cases were excluded from the study if the dog had received additional treatments aimed at curing the MGT, although dogs that received palliative treatments, such as anti-inflammatories or antibiotics, were included.

Histological sections were evaluated from all cases to confirm the diagnosis of malignant MGTs, and neoplasms were subclassified according to the classification scheme proposed by Goldschmidt et al. [14].

Immunohistochemistry was performed to detect p53 and p16 as previously described [15,16]. A mouse anti-human p53 clone pAb 240 antibody (BD Biosciences, San Jose, CA) was used to detect p53. This was used at a dilution of 1:100 with a canine osteosarcoma positive control. A mouse anti-human p16 clone G175-405 antibody (BD Biosciences) at a 1:25 dilution was used to detect p16. A canine oral SCC that had been previously found to demonstrate intense p16 immunostaining [16] was used as the positive control. Slides for which the primary antibody was omitted were used as negative controls for both antibodies.

Canine MGTs were classified as p53-positive if 20% or more of the cells contained nuclear immunostaining and as p53-negative if less than 20% of the neoplastic cells demonstrated p53 immunostaining. The MGTs were classified as p16-positive, p16-negative, or p16-intermediate. Neoplasms that were p16-positive had intense nuclear and cytoplasmic immunostaining in over 80% of the neoplastic cells. Neoplastic cells within p16-negative MGTs did not contain any significant p16 immunostaining, while p16-intermediate MGTs had low to moderate intensity of p16 immunostaining that was confined to the cytoplasm in over 10% of the cells. The thresholds used to classify the immunostaining for both antibodies were based on the thresholds used most frequently in humans, and more limitedly, in veterinary literature.

Differences between groups were investigated by Pearson chi-squared or Fisher’s exact tests and survival times were investigated using Kaplan–Meier and Cox regression analyses using IBM SBSS Statistics v25 (IBM Incorporated, Armonk, NY, USA).

## 3. Results

### 3.1. Cases Included and Histological Evaluation of MGTs

A total of 35 malignant MGTs were included in this study. These included five that were diagnosed in 2012, 12 in 2013, 8 in 2014, and 10 in 2015. The MGTs were subclassified into 8 different histological types. The tumors included 17 simple carcinomas, which were further subclassified into tubular, tubulopapillary, cribriform, and cystic papillary carcinomas (Table 1).

### 3.2. Immunohistochemistry Evaluation

**Figure 1:**
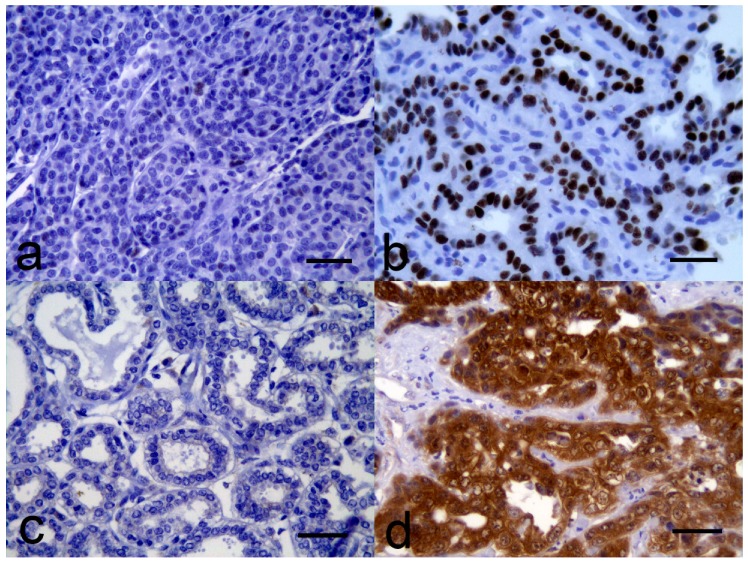
Canine malignant mammary gland neoplasms: (**a**) No significant immunostaining for p53 is visible within the neoplastic cells; (**b**) Intense nuclear p53 immunostaining is visible within most neoplastic cells; (**c**) While a proportion of stromal cells contain weak intracytoplasmic p16CDK2A immunostaining, immunostaining is absent from the neoplastic cells. (**d**) Intense intranuclear and intracytoplasmic p16^CDK2A^ immunostaining is visible within almost all of the neoplastic cells. All bars = 50 µm.

A total of 28 of 35 (80%) MGTs were p53-negative (Figure 1a), while 7 of 35 (20%) MGTs were p53-positive (Figure 1b). Immunostaining within the MGTs was biphasic so that either most of the cells had intense nuclear immunostaining or scattered cells had faint nuclear immunostaining. Therefore, while a 20% cutoff was used, all the p53-positive MGTs had a much higher proportion of cells showing nuclear immunostaining. Of the 11 histological types of MGTs, the p53-positive neoplasms were spread among five types, and there was no significant association between p53 positivity and the histological type of MGT (*p* = 0.73).

Of the 35 canine malignant MGTs, 28 (80%) were p16-intermediate and were characterized by showing a pattern and intensity of immunostaining that was similar to the immunostaining within surrounding non-neoplastic epithelium. In contrast to the p16-positive MGTs, immunostaining in the p16-intermediate MGTs was strictly cytoplasmic. Five (14%) MGTs were p16-negative (Figure 1c), and two (6%) were p16-positive (Figure 1d). Interestingly, both solid carcinomas in the study had decreased p16 immunostaining; however, there was no significant association between p16 immunostaining and the histological type of MGT (*p* = 0.16).

Canine MGTs that were p53 positive were significantly more likely to have altered p16 immunostaining, with only 2 of 7 (29%) p53-positive MGTs being classified as p16-intermediate. In contrast, 26 of 28 (93%) of p53-negative MGTs were classified as p16-intermediate (*p* = 0.001).

### 3.3. Survival of Dogs with MGTs

A total of 19 (54%) dogs died of their MGTs in this study. Seven dogs died of other causes, and nine dogs were still alive in January 2018. Of the dogs that remained alive at the end of the study, the minimum follow-up time was 807 days after diagnosis. There were no significant differences in survival rates between the different histological types of mammary gland neoplasms (*p* = 0.17). Of the dogs with p53-positive MGTs, 4 of 7 (57%) died of their MGTs. This was not significantly different from the dogs with p53-negative MGTs of which 15 of 28 (54%; *p* = 0.6) died due to their MGTs. A total of 50% of dogs with p16-intermediate and p16-positive MGTs died due to their MGTs. While 4 of 5 (80%) of dogs with p16-negative MGTs died due to mammary cancer, this difference was not statistically significant (*p* = 0.46). When MGTs with altered p16 immunostaining (either increased or decreased) were considered as a group, 5 of 7 (71%) of these dogs died due to their MGTs, which was not significantly higher than dogs with p16-intermediate MGTs (50%; *p* = 0.28).

The overall estimated mean survival time (MST) of the 35 dogs with malignant MGTs in this study was 882 days (95% CI 694–1071 days; Table 2). Dogs that had MGTs that were p53-positive had an MST of 670 days (95% CI 355–986 days), which was not significantly different from the MST of dogs with p53-negative MGTs (934 days; 95% CI 723–1144 days, *p* = 0.57). There were no significant differences in the MST of dogs with p16-positive MGTs (307 days; 95% CI 63–551 days), p16-negative MGTs (683 days; 95% CI 441–926 days), or p16-intermediate MGTs (927 days; 95% CI 704–1150 days; *p* = 0.31). When dogs with p16-positive or p16-negative MGTs were considered as a single group, these dogs had a MST of 620 days (95% CI 372–864), which was not significantly shorter than dogs with p16-intermediate MGTs (927 days; 95% CI 704–1150, *p* = 0.33).

To reduce the potential confounding effect of comparing immunostaining in types of carcinomas that have previously been shown to have different survival times, associations between immunostaining and survival were evaluated in just the 13 dogs with simple carcinomas. In these dogs, the MST of the three dogs with p53-positive simple carcinomas (564 days; 95% CI 73–1055) was not significantly different from the MST of the 10 dogs with p53-negative carcinomas (779 days; 95% CI 580–977; *p* = 0.49). Likewise, the MST of the dog with the p16-positive simple carcinoma (131 days) was not significantly different from the MST of the three dogs with p16-negative (854 days; 95% CI 621–1087; *p* = 0.08) or the 9 dogs with p16-intermediate carcinomas (748 days; 95% CI 511–985; *p* = 0.07).

## 4. Discussion

Immunostaining to detect p53 did not predict which dogs were more likely to die of their MGTs in this study. Furthermore, no significant difference in MST between dogs with p53-positive and p53-negative MGTs was detected. These results are in contrast to a study of 10 dogs with MGTs that reported that p53 positivity significantly predicted a shorter survival time [5]. However, the results of the present study are consistent with a study of 40 MGTs that also did not detect a significant association between p53 immunostaining and survival [6]. Considering the important role that p53 plays in maintaining genetic stability within a cell, the lack of prognostic significance is perhaps surprising. However, as the present study contained relatively small numbers of MGTs, it is possible that studies of larger samples could reveal a significant association between MGT behavior and p53 immunostaining. In addition to the small total number of MGTs, this study also contained many different MGT types. The inclusion of small numbers of numerous MGT types could be expected to increase variability of the data, which may have obscured a significant role of p53 in predicting prognosis. Studies with larger numbers of a single MGT type are required to definitively exclude a role of p53 in predicting the prognosis of a canine MGT.

Alternatively, it is possible that the difficulty of using immunostaining to differentiate between normal p53 function and loss of p53 function due to nonsense mutations in the p53 gene prevented p53 immunostaining from being associated with prognosis. The disadvantages of p53 immunostaining can be avoided by molecular techniques. In a study of 42 canine MGTs, the p53 gene was sequenced, and the presence of p53 gene mutations was associated with neoplasm recurrence and death due to the MGT [17]. In addition, the level of gene expression has been evaluated in canine MGTs and has revealed that differences in the level of p53 expression were present between MGTs of different grades. Additionally, p53 expression was predictive of developing nodal metastases [18,19]. It is possible that p53 sequencing or quantification of expression could be a useful prognostic marker for canine MGTs; however, these assays are technically challenging and less suitable for use in a routine laboratory setting.

In the present study, 20% of the MGTs were p53-positive. This is consistent with previous studies that reported p53 immunostaining in 3 of 10 (30%) [5], 14 of 86 (16.3%) [4], and 12 of 40 (30%) [6] canine MGTs. Evidence from previous studies, as well as the present study, suggests that around one in five canine MGTs contains missense mutations in the p53 gene. However, the presence of these mutations does not appear to greatly influence the behavior of the neoplasm, and most evidence from canine and human studies suggest that p53 immunostaining may be, at best, a weak marker of prognosis for canine MGTs.

There were no significant differences in the survival rates or the MSTs between dogs with MGTs that were p16-negative, p16-intermediate, or p16-positive. However, a potential role of p16 as a prognostic marker cannot definitively be excluded, as dogs with p16-negative MGTs had the lowest survival rates and there appeared to be a trend for dogs with either p16-negative or p16-positive MGTs to survive for a shorter time than dogs with p16-intermediate MGTs. Similarly, in humans, breast cancers that were p16-positive were reported to have a less favorable prognosis. It is possible that significant differences between the groups were not detected in the present study because of the small number of either p16-negative or p16-positive MGTs that were included. The small number of p16-negative MGTs in the present study (14%) was unexpected, as a previous study had detected loss of p16 immunostaining in 24 of 79 (30%) of canine MGTs [13]. In the previous study, neoplastic cells with intense nuclear and cytoplasmic p16 immunostaining were not reported [13].

In the present study, three distinct patterns of p16 immunostaining were recognized in canine MGTs. The most common pattern consisted of faint to moderate immunostaining that was confined to the cytoplasm. As this p16-immunostaining pattern is similar to that seen in normal epithelium, this staining pattern was considered to represent an intact pRb-p16 pathway. The high proportion of MGTs with this immunostaining pattern suggests that most neoplasms develop without developing mutations within the pRb-p16 pathway. The second pattern was seen in five MGTs and consisted of an absence of p16 immunostaining within the neoplastic cells. This pattern is consistent with the presence of mutations within the p16 gene preventing protein production. Finally, the least common pattern was intense cytoplasmic and nuclear immunostaining in almost all neoplastic cells. Evidence from human oral SCCs suggests that these neoplasms had mutations that prevented the production of pRb [9]. As p16-intermediate staining was interpreted as a normal pRb-p16 pathway, it was hypothesized that both increased and decreased p16 was indicative as a disruption in this cell regulation pathway and that this disruption could be prognostic. However, no differences in survival rates or MST were observed when MGTs with ‘normal’ pRb-p16 function were compared with those with altered pRb or p16 proteins. Overall, while p16 immunostaining has been found to be strongly prognostic in some types of human and veterinary neoplasms [10,20], the present study revealed little evidence to support the use of p16 immunostaining as a prognostic marker for canine MGTs. However, it remains possible that the evaluation of larger numbers of canine MGTs, especially series of individual types of MGTs, may reveal p16 to be prognostically significant in these tumors.

Canine MGTs with missense mutations in p53 were significantly more likely to also have mutations that disrupted the normal pRb-p16 pathway. It is uncertain why mutations in p53 would be predisposed to additional mutations in pRb or p16; however, the results of the immunostaining in this study suggest that most MGTs develop without missense mutations in p53 or a disrupted pRb-p16 pathway. A smaller subset of MGTs develop as a result of disruption to both pRb-p16 and p53.

In the present study of 35 canine malignant MGTs, the histological type was not found to be prognostic. This is in contrast with an earlier study of 229 canine MGTs that revealed a prognostic significance of histological type [2]. In the present study, comparatively small numbers of samples were included and the high number of different MGT types meant that only one or two examples of some types were included. It appears most likely that the lack of prognostic significance of MGT type observed in the present study was due to the relatively small number of total neoplasms included and the high heterogeneity of the MGTs in this study.

As significant differences in survival times between MGTs of different types have been reported [2], the prognostic significance of p53 and p16 was evaluated in only a single carcinoma type. Simple carcinomas were used for this analysis as these were the most common MGT type within the present study. When just simple carcinomas were considered, there were no significant differences in survival times of dogs with MGTs that had different immunostaining characteristics. However, as only 13 simple carcinomas were included in the study, the ability of these antibodies to predict prognosis cannot be excluded.

For a prognostic marker to be clinically useful, it has to be shown to consistently predict prognosis when used by multiple different pathologists in multiple different diagnostic laboratories. In veterinary pathology, many prognostic grading schemes and other markers have been proposed, but few have been shown to consistently predict prognosis when widely used [21]. A common reason for low consistency is the inter-pathologist variability when subjectively evaluating histological criteria [22]. A potential advantage of immunohistochemistry is the comparative ease of interpretation when compared with features such as cell differentiation. Unfortunately, while both p53 and p16 immunostaining were easy to interpret in the present study, neither significantly predicted prognosis.

## 5. Conclusions

The results of this study show that canine MGTs demonstrate variable p53 and p16 immunostaining. However, these variations do not appear to influence the biological behavior of the neoplasm sufficiently to significantly alter survival rates or MSTs of the affected dogs. This study is the third to investigate p53 immunostaining as a prognostic marker in canine MGTs and the second to suggest that p53 may not be strongly predictive of prognosis for these neoplasms. This is the first time that p16 has been evaluated as a prognostic marker for canine MGTs.

## Figures and Tables

**Table 1:** Summary of p53 and p16CDKN2A protein (p16) immunostaining observed within the different histological types of canine malignant mammary gland neoplasms.

Histological Subtype	Total	p53-pos	p53-neg	p16-pos	p16-int	p16-neg
Simple carcinomas (total)	13	3	10	1	9	3
Tubular carcinoma	6	2	4	0	4	2
Tubulopapillary carcinoma	3	1	2	1	1	1
Cribriform carcinoma	2	0	2	0	2	0
Cystic papillary carcinoma	2	0	2	0	2	0
Mixed mammary carcinoma	6	0	6	0	6	0
Intraductular papillary carcinoma	6	2	4	1	5	0
Complex carcinoma	3	1	2	0	3	0
Ductular carcinoma	3	0	3	0	3	0
Solid carcinoma	2	1	1	0	0	2
Comedocarcinoma	1	0	1	0	1	0
Adenosquamous carcinoma	1	0	1	0	1	0
**All types**	**35**	**7**	**28**	**2**	**28**	**5**

**Table 2:** Survival times of dogs with malignant mammary gland neoplasms.

	Number	Estimated Mean Survival Time (95% CI) Days	*p* Value
All tumors	35	882 (694–1071)	
p53 status			0.57
Positive	7	670 (355–986)	
Negative	28	934 (723–1144)	
p16^CDKN2A^ status			0.31
Intermediate	28	927 (704–1150)	
Negative	5	683 (441–926)	
Positive	2	307 (63–551)	
